# Safety and anti-hyperglycemic efficacy of various tea types in mice

**DOI:** 10.1038/srep31703

**Published:** 2016-08-17

**Authors:** Manman Han, Guangshan Zhao, Yijun Wang, Dongxu Wang, Feng Sun, Jingming Ning, Xiachun Wan, Jinsong Zhang

**Affiliations:** 1 State Key Laboratory of Tea Plant Biology and Utilization, School of Tea & Food Science, Anhui Agricultural University, Hefei, Anhui 230036, PR China

## Abstract

Tea, a beverage consumed worldwide, has proven anti-hyperglycemic effects in animal models. Better efficacies of tea beverages are frequently associated with high-dose levels, whose safety attracts considerable attention. Based on the inherent nature of tea catechin oxidation, fresh tea leaves are manufactured into diverse tea types by modulating the oxidation degree of catechins. The present study aimed to assess various tea types for their safety properties and anti-hyperglycemic effects. Mice were allowed free access to tea infusion (1:30, w/v) for one week, and the rare smoked tea caused salient adverse reactions, including hepatic and gastrointestinal toxicities; meanwhile, the widely-consumed green and black teas, unlike the rare yellow tea, suppressed growth in fast-growing healthy mice. When mice were fed a high-fat diet and allowed free access to tea infusion (1:30, w/v) for 25 days, only yellow tea significantly reduced blood glucose. Therefore, various teas showed different safety profiles as well as anti-hyperglycemic efficacy strengths. To achieve an effective and safe anti-hyperglycemic outcome, yellow tea, which effectively suppressed high-fat diet-induced early elevation of hepatic thioredoxin-interacting protein, is an optimal choice.

Tea made from the plant *Camellia sinensis* L. is one of the most commonly consumed beverages worldwide, and has been considered a crude medicine for over 4000 years in China, the origin of tea^1^. Teas are grossly classified into six categories according to fermentation, which causes the oxidation of tea catechins found with high abundance in fresh tea leaves, through wet-heat conditions and/or intrinsic polyphenol oxidases and peroxidases. They are: non-fermented green tea; mildly-fermented white and yellow teas; semi-fermented oolong tea, wherein pronounced catechin oxidation occurs only on the rim of tea leaves; intensively-fermented black and dark teas^2^. Diverse catechin oxidation products formed during non-enzymatic and/or enzymatic oxidation processes confer a unique color to each tea type, and significantly contribute to tea characteristic flavor, making it popular either worldwide (e.g. black tea) or only in certain regions.

In the past two decades, catechins extracted from green tea, particularly purified epigallocatechin-3-gallate (EGCG), which accounts for more than half of total catechins and is the most biologically active green tea catechin, have been demonstrated to be effective in alleviating metabolic syndrome, reducing neurodegenerative disease incidence, and cancer prevention^3^. Accordingly, green tea catechin extracts or EGCG are gaining popularity for the prevention of chronic diseases. On the other hand, multiple medicinal foods and food supplements are not always as safe as generally assumed, and long-standing experience in traditional remedies does not necessarily guarantee safety^4^,^5^. The effectiveness of tea catechins from a pharmacological perspective depends on dosage. It is known that the efficacious EGCG doses used in animal experiments, e.g. for treating carcinogen-induced tumors or high-fat-evoked metabolic syndrome, are very close to toxic levels^6^,^7^,^8^. The main side effects of EGCG or green tea catechin extracts found in humans and experimental animals are hepatotoxicity and hemorrhagic lesions in the stomach and intestine^9^,^10^,^11^,^12^,^13^,^14^. Interestingly, a recent report with extensive social impact showed that high-dose green tea polyphenols severely affect development and reproduction in *Drosophila melanogaster*^15^.

Compared to green tea, other teas (except for black tea) that undergo different degrees of fermentation have not been extensively studied for their health benefits. Limited comparative studies using different tea types only focused on efficacy, without considering the other side of the coin, namely, safety. Do all teas provide equal health benefits? The present study addressed this issue, focusing on safety and efficacy. A total of four tea types purchased from the market were compared for safety profiles in healthy mice and anti-hyperglycemic efficacy in mice fed a high-fat diet. Compared to commonly consumed green or black tea, a rare tea assessed, herein termed as “smoked tea” (distinguished from other tea types by prolonged exposure of tea leaves to smoke), had a lower safety profile; meanwhile, the rare yellow tea made from coarse old leaves exhibited a unique feature of higher safety and better efficacy.

## Results

### Chemical compositions of various tea infusions

There was a wide distribution range of both total catechin (0.10−4.33 mg/mL) and EGCG (0.04−2.52 mg/mL) amounts in the four tea infusion types (1:30, w/v) examined. The order of either total catechin or EGCG levels was green tea > smoked tea > yellow tea > black tea, and EGCG always accounted for the largest catechin proportion (Table 1). Lower levels of caffeine and theanine, and higher levels of either soluble sugars or polysaccharides of yellow tea infusion were remarkable (Table 2); these distinct differences are consistent with the fact that the yellow tea assessed was manufactured from coarse old tea leaves^16^,^17^, which otherwise are discarded since they are not to suitable for high-quality tea production, as for flavor and taste.

### Short-term tolerance of mice to tea infusion

**Figure 1:**
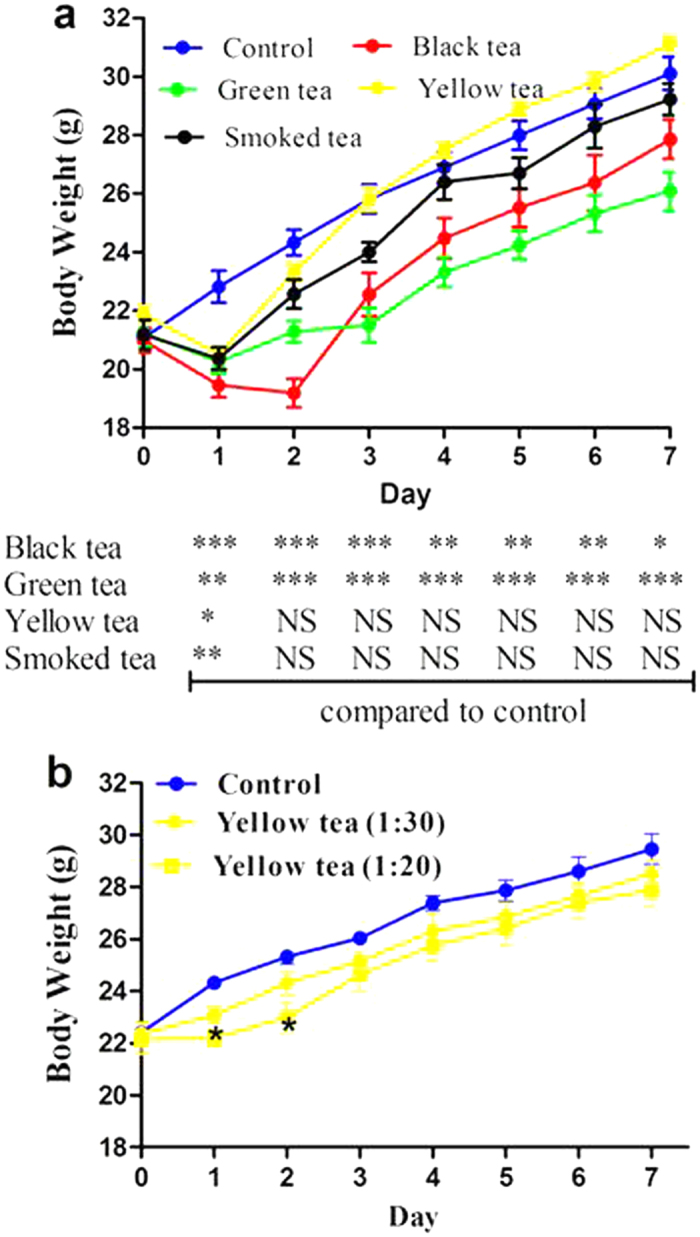
Body weights of mice consuming water or tea infusion. Mice were allowed free access to water (control group) or tea infusion and regular diet for one week. (**a**) Comparison of different teas (1:30, w/v). (**b**) Dose effect of yellow tea. Data are presented as mean ± SEM (n = 6 or 7). Compared to control, *P < 0.05, **P < 0.01 and ***P < 0.001. NS, non-significant.

Mice were fed a regular diet with free access to water as control or tea infusion (1:30, w/v) for one week. The first day of tea drinking, mice were not familiarized with tea location, irrespective of its type, as evidenced by body weight loss (Fig. 1a); however, mice in yellow and smoked tea groups rapidly recovered growth afterward without further significant differences in body weights compared with controls. Noticeably, significant growth suppression was persistently observed throughout the entire experimental period in mice consuming green or black tea (Fig. 1a). This study investigated the tolerance of mice to tea infusion, at a dosage equivalent to high-dose consumption in humans, for subsequent anti-hyperglycemic studies. It was terminated on day 7. During dissection, a strong bad smell was released from the peritoneal cavity of smoked tea group mice, but not from other groups; this unpleasant odor was associated with darkened intestines in these mice (Table 3). Consistently, serum hepatic enzymes were significantly increased only in mice consuming the smoked tea (Table 3). The four types of tea did not affect lipid profile or renal function: serum levels of triglyceride, total cholesterol, blood urea nitrogen and creatinine remained normal (data not shown). Interestingly, the bad smell and darkened intestine were still observed in the smoked tea group after two weeks (data not shown), and salient growth suppression did not occur in mice consuming higher concentrations of yellow tea infusion (1:20, w/v) (Fig. 1b). Overall, the rare smoked tea raised much safety concern, while the rare yellow tea at high levels appeared to be safer than the widely-consumed green and black teas, regarding growth suppression of fast-growing healthy mice.

### Anti-hyperglycemic effects of different tea infusions in mice fed a high-fat diet

**Figure 2:**
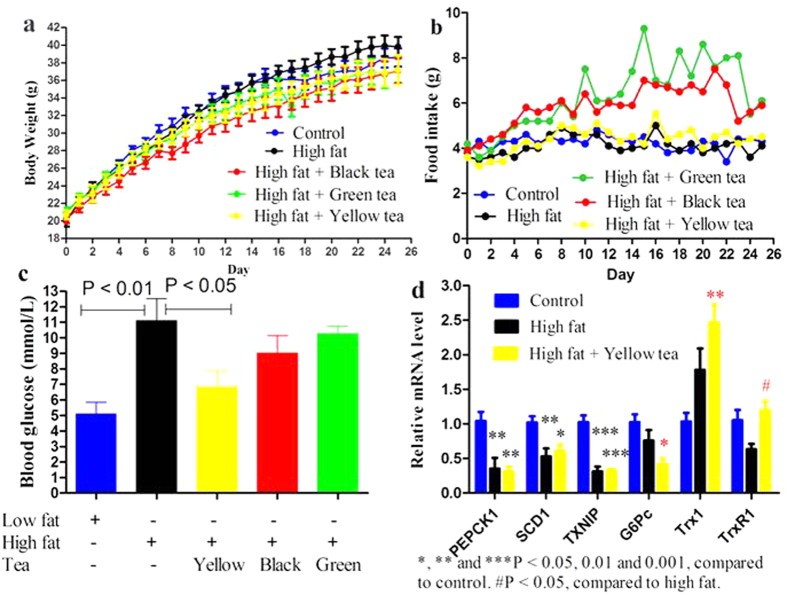
Effects of different teas on high-fat diet induced metabolic dysfunction. Mice were allowed free access to water and regular diet (control group), water and high-fat diet as model (high fat group) or tea infusion (1:30, w/v) and high-fat diet (experimental group) for 25 days. (**a**) Body weights. (**b**) Food intake. (**c**) Fasting blood glucose levels. (**d**) Hepatic genes responsible for or associated with gluconeogenesis and lipogenesis. Data are presented as mean ± SEM (n = 6 or 7).

Smoked tea was excluded from subsequent functional experiments for potential toxic reactions. Mice were fed a regular diet (control group) or high-fat diet for 25 days. Compared with the control diet, high-fat diet caused no further body weight increase, and green, black and yellow teas showed no effects on body weight in mice fed a high-fat diet (Fig. 2a). Average food intake was recorded daily, and not affected by yellow tea. However, green and black teas overtly increased food intake from day 5 to 25, particularly during the period from day 12 to 22, with a more pronounced effect for green tea (Fig. 2b). According to total food intake or area under curve of food intake, green and black teas increased food intake by more than 50% and 40%, respectively. Such an unusual phenomenon observed in mice of green and black tea groups, respectively, was not caused by feeding environments since normal intake was obtained in other groups, or by erroneous actions that resulted in chow becoming debris, which would constitute a confounding factor. Indeed, we did not perceive food particles in animal bedding in any experimental group. Whether tea consumption increases appetite has not been well elucidated. The present uninterrupted daily observations that are different from most studies assessing weekly food intake suggested that one should remain vigilant until this issue is unambiguously clarified. Although high-fat diet did not further increase body weight, it substantially elevated fasting blood glucose levels, compared to control values (Fig. 2c). Intriguingly, only yellow tea but not green and black teas significantly prevented this elevation. We thus examined high fat-induced alterations of hepatic genes responsible for or associated with gluconeogenesis and lipogenesis, and the corresponding effects of yellow tea on these genes, including phosphoenolpyruvate carboxy-kinase 1 (PEPCK1), glucose-6-phosphatase, catalytic subunit (G6Pc), thioredoxin-interacting protein (TXNIP), fatty acid synthase (FASN), acetyl-coenzyme A carboxylase alpha and beta (ACCα and ACCβ), stearoyl-coenzyme A desaturase 1 (SCD1), forkhead box O1 (Foxo1), sterol regulatory element-binding protein 1 (Srebp1), thioredoxin 1 (Trx1) and Trx reductase 1 (TrxR1). High-fat diet paradoxically resulted in downregulation of the hepatic genes PEPCK1, SCD1 and TXNIP (Fig. 2d), reminiscent of metabolic adaption to high fat or obesity as documented in other studies^18^,^19^,^20^,^21^. Under these circumstances, yellow tea did not disrupt the adaptive responses; it appeared to improve transcriptional adaption as evidenced by G6Pc (Fig. 2d). Yellow tea significantly increased Trx1 mRNA and effectively prevented TrxR1 mRNA from high-fat diet induced downregulation (Fig. 2d). High-fat diet accompanied with or without yellow tea did not influence other genes examined or the hepatic glycogen, triglyceride and non-esterified fatty acid levels (data not shown). Taken together, in contrast to green or black tea, yellow tea suppressed elevation of fasting blood glucose without promoting food intake, at least in part by enhancing hepatic transcriptional adaption to high-fat diet and upregulating the hepatic genes involved in the Trx system that participates in positive feedback of AMPK^22^,^23^,^24^, a paramount energy metabolism sensor.

### Profile of transcriptional adaption to extended or shortened feeding periods with high-fat diet and effects of yellow tea

**Figure 3:**
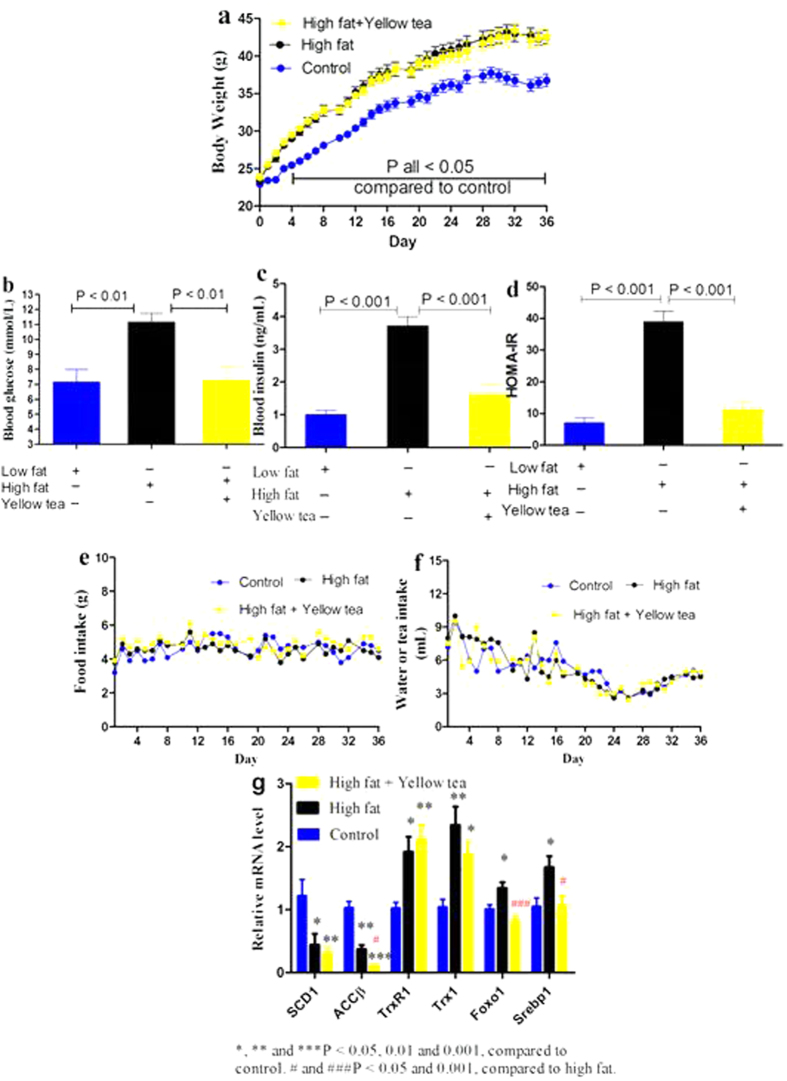
Effects of yellow tea on high-fat diet induced metabolic dysfunction. Mice were allowed free access to water and regular diet (control group), water and high-fat diet as model (high fat group) or yellow tea infusion (1:30, w/v) and high-fat diet (yellow tea group) for 36 days. (**a**) Body weights. (**b**) Fasting blood glucose levels. (**c**) Fasting blood insulin amounts. (**d**) HOMA-IR, which is calculated according to the formula: glucose (mmol/L) × insulin (mU/L) ÷ 22.5. (**e**) Food intake. (**f**) Water or tea consumption. (**g**) Hepatic genes responsible for or associated with gluconeogenesis and lipogenesis. Data are presented as mean ± SEM (n = 8).

**Figure 4:**
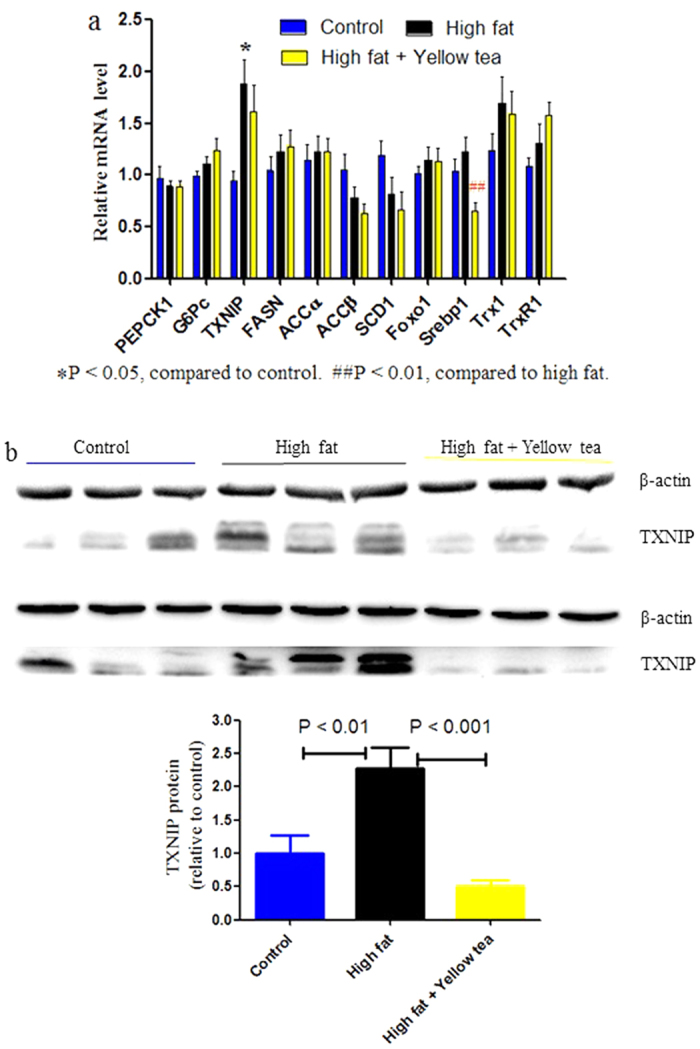
Effects of short-term high-fat feeding on hepatic genes and a protein responsible for or associated with gluconeogenesis and lipogenesis. Mice were allowed free access to water and regular diet (control group), water and high-fat diet as model (high fat group) or yellow tea infusion (1:30, w/v) and high-fat diet (yellow tea group) for 9 days. (**a**) Hepatic genes responsible for or associated with gluconeogenesis and lipogenesis. (**b**) Hepatic TXNIP protein. Data are mean ± SEM (a, n = 8; b, n = 6).

To further characterize the intriguing transcriptional adaption found above and explore yellow tea effects, we extended the feeding time with high-fat diet to 36 days. High-fat diet significantly increased body weight compared to control; however, yellow tea had no effect on body weight gain enhanced by high-fat diet (Fig. 3a). Nonetheless, high-fat diet-induced elevations in fasting blood glucose and insulin as well as homeostasis model assessment of insulin resistance (HOMA-IR) were effectively suppressed by yellow tea (Fig. 3b–d). Yellow tea had no effect on food intake or water consumption (Fig. 3e,f). Along with the detrimental increase of hepatic Foxo1 and Srebp1, high-fat diet also induced benign transcriptional adaption evidenced by reduced SCD1 and ACCβ mRNAs and increased TrxR1 and Trx1 mRNAs levels (Fig. 3g). Yellow tea again didn’t interfere with such adaptive responses, and seemed to enhance the response by further decreasing ACCβ mRNA amounts; more importantly, it significantly prevented Foxo1 and Srebp1 from the unwanted elevation triggered by high-fat diet (Fig. 3g). Notably, high-fat diet with or without yellow tea did not affect other genes including PEPCK1, G6Pc, TXNIP, FANS and ACCα or glycogen and triglyceride levels in the liver. However, high-fat diet-induced slight but significant (P < 0.05) elevation in hepatic non-esterified fatty acid was significantly (P < 0.05) suppressed by yellow tea to normal levels (data not shown). To evaluate whether the mice could rapidly display an adaptive response, we shortened the feeding time of high-fat diet to 9 days. Hepatic transcriptional adaption did not occur in the short-term. Increased hepatic TXNIP mRNA in the high-fat group but not the yellow tea intervention group as well as significantly reduced hepatic Srebp1 mRNA amounts in the intervention group (Fig. 4a) implied that yellow tea at the earlier stage could have imparted beneficial effects on the liver undergoing initial irritation by high fat. Most importantly, high-fat diet caused a rapid hepatic TXNIP protein elevation, which was significantly reversed by yellow tea (Fig. 4b). See Supplementary Fig. 1 for non-cropped immunoblots. Based on this finding, we further evaluated TXNIP protein expression in the hepatic tissues of 25-day and 36-day feeding experiments. As shown in Supplementary Figs 2 and 3, compared with normal control, mice exposed to high-fat diet displayed significantly reduced protein levels, consistent with experiments suggesting transcriptional adaptation. Further, yellow tea did not interfere with benign TXNIP protein alteration.

## Discussion

The present study showed that various teas have different safety profiles and anti-hyperglycemic effects. Importantly, the yellow tea known as Huang-Da-Cha, exhibited higher safety and better anti-hyperglycemic efficacy.

A recent clinical trial indicated that daily intake of only 800 mg (400 mg twice daily) green tea catechin extracts for half a year causes hepatotoxicity in many participants^12^. The maximum tolerated dose, defined as the amount causing 25% dose-limiting toxicity was determined to be 1200 mg (600 mg twice daily) of such extracts for six months in another clinical trial^11^. Green tea polyphenols at high doses needed for life extension, however, caused developmental retardation and impaired reproduction in *Drosophila melanogaster*^15^. These negative effects triggered a debate over the safety of tea catechins^25^,^26^,^27^. Green tea consumption has a long history of proven safety. The pronounced discrepancy in toxicity between consumption of green tea catechin extracts and green tea itself may be caused by the beneficial effects of other ingredients in green tea on the pro-oxidant effects of high-dose catechins, a major mechanism involved in the toxicity of tea catechins^10^. In addition to tea catechins, green tea also contains L-theanine and polysaccharides, which are well known for hepatoprotective activity^28^,^29^,^30^,^31^,^32^,^33^.

To overcome the unpredictable side effects occurring with green tea catechin extracts or EGCG, and mimic the human consumption style of tea drinking, the present study employed hot water infusion of tea and allowed mice free access to tea infusion. Most people consume 5 to 10 g of tea daily. However, a few individuals are accustomed to drinking large amounts of tea, as high as 20 g per day. The present study in mice used 1/30 tea infusion, which is a little lower than the dose (1/20 green tea infusion) used to improve glucose tolerance in rats exposed to high-fat diet^34^. Based on a Km of 3 for mice and 37 for humans^35^, and cross-sectional body weight of 33 g (Fig. 3a) and representative daily tea consumption of 4 mL (Fig. 3f), the dose translation from a mouse consuming 1/30 tea infusion to a human with an average body weight of 65 kg is 21 g per day. On the other hand, peak plasma concentrations of EGCG in mice and humans following pharmacological oral doses of EGCG are nearly equivalent, at 2 to 9 μM and 7.5 μM, respectively^36^. Therefore, the dose used here corresponds to high-dose levels consumed by humans. Our previous studies consistently showed that growth suppression in fast-growing mice was involved in EGCG-triggered toxic reactions; accordingly, growth suppression in normal mice should be considered an adverse reaction^9^,^10^. This concept is consistent with the finding that high-dose green tea polyphenols halt the development of *Drosophila melanogaster*^15^. EGCG-triggered acute growth inhibition is associated with suppression of growth hormones, including testosterone, estradiol, leptin, insulin and insulin-like growth factor I^37^. Furthermore, only EGCG, but not other tea catechins, suppressed rat growth and plasma levels of growth hormones^37^, suggesting that diverse oxidized products of tea catechins formed in different tea types do not suppress animal growth similarly. We found that the growth-suppressive effect of green and black teas was identical, and more robust than either yellow or smoked tea.

To our great surprise, infusion of smoked tea, which is baked to dryness by charcoal fire under a heavy-coated wet and compressed environment, caused not only hepatotoxicity but also gastrointestinal abnormalities. The levels of EGCG and tea catechins of smoked tea infusion were nearly equivalent to those found in green tea infusion. It is plausible that certain exogenous compounds, such as polycyclic aromatic hydrocarbons deposited on tea leaves exposed to wood combustion fumes, may render some tissues susceptible to high-dose EGCG and tea catechins. It is worth noting that several kinds of smoked black tea that originated from Taiwan and south-eastern China contain much high levels of benzo(a)anthracene and chrysene compared with non-smoked black tea^38^. On the other hand, healthy mice were more tolerant to yellow tea infusion compared with green tea beverage in growth suppression. Such a high safety profile of yellow tea would be attributed to reduced EGCG and tea catechin contents as well as increased tea polysaccharides, which might be helpful for quenching high-dose EGCG-initiating ROS. EGCG has either antioxidant or pro-oxidant properties depending upon dose levels and environments, whereas tea polysaccharides are known only for their antioxidant activity irrespective of dose levels^39^. Both smoked and yellow teas used herein are rare tea species; however, they may be two paradigms showing that exogenous inclusions with uncertain safety record would lower the threshold of potential toxicity risk of copious consumption of tea. This threshold could be increased by optimizing the constitution of endogenous functional components. It is worth noting that all the toxicity concerns are attributed to high doses necessary to achieve pronounced efficacy. Tea as a beverage is normally consumed at less than 10 g daily, whereas at least 20 g tea per day is needed to yield anti-hyperglycemic effect. Therefore, the present findings regarding smoked tea do not automatically imply that this traditional beverage is associated with adverse effects; as Paracelsus, the founder of toxicology, puts it, the dose makes the poison. Nonetheless, the current findings suggest the need for rigorous risk evaluation of smoked teas.

Tea has many beneficial functions, among which alleviation of metabolic syndrome has attracted attention in recent years. There is a high likelihood that enhanced safety incurs compromised drug efficacy. We thus compare anti-hyperglycemic effects of yellow, black and green teas. The latter two tea types have been extensively investigated independently or comparatively, concerning metabolic syndrome improvements. Most studies showed that black tea, with catechins undergone pronounced oxidation, still yields an efficacy almost identical to green tea with abundant tea catechins^40^,^41^,^42^, suggesting lower levels of tea catechins in yellow tea do not attenuate functions. Employing short-term models to assess the effect of tea on metabolic syndrome should be encouraged because high-dose tea is required in such investigations. For example, 4-week administration of diet containing 1% EGCG reduced high-fat diet-induces obesity in mice^7^; however, 6-week administration of diet containing the same amount of EGCG caused pro-inflammatory responses in mice^8^. Fasting blood glucose levels of ICR mice subjected to high-fat diet treatment for only three weeks were shown to increase by 120%^43^. Following this model, we found that fasting blood glucose levels of ICR mice fed a high-fat diet for 25 days were significant elevated by 118%; only yellow tea but not the black or green one significantly prevented this elevation. On the contrary, both black and green teas but not the yellow one overtly promoted food intake during the latter half period in this short-term intervention study. Hence, it is encouraging that safety and anti-hyperglycemic effects are not contradictory, but consistent in this unique yellow tea, which is manufactured from the so-called coarse old tea leaves using two special processes: (1) sealed yellowing, which allows catechin oxidation but at a slower speed, and (2) intensive heating to produce caramel flavor through Maillard reaction. The anti-hyperglycemic effect of yellow tea was further confirmed in subsequent experiments. Moreover, in the 36-day high-fat feeding model, fasting blood insulin levels and accordingly HOMA-IR of ICR mice were substantially increased, whereas yellow tea pronouncedly suppressed these effects. The anti-hyperglycemic mechanism of yellow tea was found to be associated with the suppression of high-fat diet-triggered early elevation of hepatic TXNIP. TXNIP promotes hepatic glucose synthesis and glucose release from hepatocytes; meanwhile, hepatic overexpression of TXNIP transgene in wildtype mice results in elevated serum glucose levels^44^. TXNIP suppresses cellular glucose uptake^45^ probably by inducing glucose transporter internalization via clathrin-coated pits^46^. Coincidently, coarse old tea leaves in China and Japan have been used to cure diabetes^16^,^17^. Coarse old tea leaves possess higher levels of polysaccharides than tender tea leaves with buds^16^,^17^. Multiple studies demonstrated that tea polysaccharides have anti-hyperglycemic effects^39^. Hitherto, data related to yellow tea, also known as Huang-Da-Cha, are scarce. A previous report showed that Huang-Da-Cha, but not other teas such as green, black, white, pu-erh and oolong teas, could prevent carbon tetrachloride from causing acute liver damage in rats^47^.

We preformed two high-fat diet experiments in the present study. Surprisingly, we found that high-fat diet did not stimulate body weight increase in the 25-day experiment (Fig. 2a), whereas body weight increased rapidly in the 36-day experiment (Fig. 3a). In these experiments, we discovered that mice generated an adaptive response to high-fat stimulation in both gene and protein levels (Figs 2d and 3g and Supplementary Figs 2 and 3). High-fat diet did not trigger body increase in the 25-day experiment, partly because of efficient adaptive response. The present study also found another unusual phenomenon, namely, green and black teas suppressed growth in control animals but not in mice exposed to high-fat diet. After ingestion, lipids undergo emulsification. Tea polyphenols are able to interact with the emulsified lipids, resulting in increased fecal lipid content^36^. Further, co-administration of butter and tea catechins increased fecal catechin levels and decreased plasma concentrations of free catechins^48^. Therefore, high fat leads to partial sequestration of tea polyphenols in fecal lipids and consequently compromises systemic bioavailability of tea polyphenols. Based on these considerations, once green or black tea suppresses growth in normal mice (Fig. 1a), the same amount of green or black tea probably does not reduce body weight in mice exposed to high-fat diet, especially when high-fat diet does not trigger body weight gain (Fig. 2a). To elucidate the potential reason underlying the interesting phenomenon, namely green and black teas promoted food intake but did not increased body weight gain in mice fed high-fat, we found that the two teas effectively suppressed plasma leptin levels and significantly promoted elevation of hepatic beta-oxidation genes (Supplementary Fig. 4). The findings suggest that increased total caloric intake was neutralized by favorable metabolic regulation by green and black tea, leading to body weight homeostasis.

The present study has several limitations that need to be addressed in additional studies. (1) The components responsible for the beneficial effects of yellow tea and the deleterious effects of smoked tea have not been identified. We assumed that moderately oxidized products of tea catechins derived from yellowing process, the abundant polysaccharides in coarse old tea leaves used for preparing yellow tea, and specific compounds that confer yellow tea a unique caramel flavor might synergistically or independently contribute to the pharmacological effects. Exogenous compounds formed during wood combustion may be a predominant factor lowering the tolerance of mice to smoked tea. (2) Toxic reactions induced by smoked tea may be attributed to necrosis, inflammation and hyperplasia in target organs. Histological investigations were not conducted to investigate these adverse effects.

In conclusion, the present study demonstrated that various teas differ in safety and anti-hyperglycemic efficacy. Rigorous risk evaluation of smoked tea is required according to the present findings. To achieve an effective and safe anti-hyperglycemic outcome, the yellow tea made from coarse old leaves, previously considered to have a lower economic value compared with popular green and black teas, is an optimal choice.

## Methods

### Chemicals

Commercial kits for alanine aminotransferase (ALT), aspartate aminotransferase (AST) and lactate dehydrogenase (LDH) activity measurements were obtained from Jiancheng Bioengineering Institute (Nanjing, China). Glucose was determined by the glucose oxidase method using a commercial kit from Huili Biotech Co., Ltd (Changchun, China). ELISA kits for measurement of insulin and leptin levels were purchased from EMD Millipore Corporation, Billerica (MA, USA). Other chemicals were of the highest grade available.

### Tea infusion

**Figure 5:**
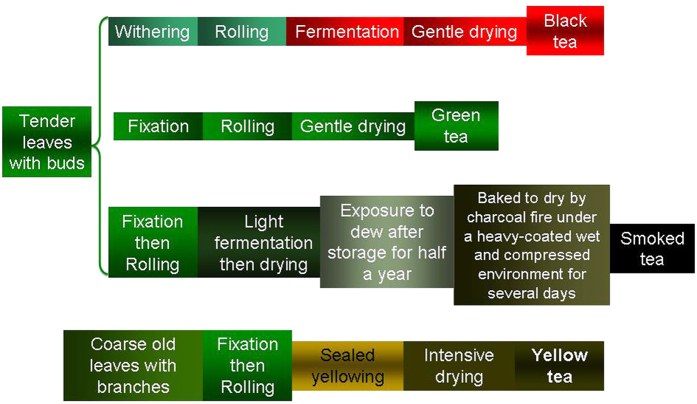


Green, black, yellow and smoked teas were purchased from the market in China. The major manufacture processes are outlined in Fig. 5. Tea infusion (1:30, w/v, unless otherwise specified) was prepared by immerging 1 g of dried tea leaves in 30 mL hot water (100 °C) for 10 min; the resultant tea drink was cooled to room temperature in a water bath, filtered, and stored at −80 °C.

### Animal and treatments

Healthy male ICR mice (20~22 g) were purchased from Shanghai SLAC Laboratory Animal Co. Ltd., and housed in a temperature (22 ± 2 °C) and humidity (40–60%) controlled environment with a 12 h light/dark cycle with free access to water or tea infusion, and regular or high-fat diet. High-fat diet (45% fat calories) and paired regular diet (10% fat calories) were purchased from TROPHIC Animal Feed High-Tech Co. Ltd, China. At the end of each experiment, mice were fasted for 12 h, anesthetized and sacrificed by cervical dislocation after peripheral blood collection from the ophthalmic vein. Serum was obtained by centrifugation at 10,000 rpm for 10 min, and stored at −80 °C until analysis. Liver tissues were excised and rinsed in ice-cold saline and then stored at −80 °C. All animals were humanely treated in accordance with the Guide for the Care and Use of Laboratory Animals (Ministry of Science and Technology of the People’s Republic of China) and all animal protocols were reviewed and approved by the ethics committee of Anhui Agricultural University. We stated the key parameters of each of the five experiments, including the route of administration, experimental period, tea infusion dose and animal number in each Figure or Table legend.

### Measurements of major chemical components of tea infusion

Catechins, theanine and caffeine were analyzed on a Waters High Performance Liquid Chromatography (HPLC) system equipped with Waters 600 controller and Waters 2489 UV Detector (280 nm). The Empower ^TM^ 3 software was used for instrument operation control and data collection. Chromatographic separation was performed on a Gemini 5u C18 110A column (250×4.60 mm, 5 μm, Phenomenex Inc., Torrance, CA), with a solvent system consisting of 0.17% (v/v) acetic acid (A) and 100% acetonitrile (B); a linear gradient at a flow rate of 1.0 mL/min was set as follows: B from 8 to 28% (v/v) in 30 min was initiated, followed by B from 28 to 100% (v/v) between 30 and 37 min, and B from 100 to 8% (v/v) between 37 and 46 min. Peaks were identified by comparison of retention times with those of standards^49^. Total sugar content of tea infusion was determined by the anthrone-sulfuric acid method^50^. Polysaccharides were isolated from tea infusion using an Amicon Ultra-0.5 Centrifugal Filter Device (UFS 503024, 3,000 Dalton cutoff).

### RNA isolation and reverse transcriptase polymerase chain reaction (RT-PCR)

Total RNA was isolated using TRIzol reagent (Takara Biotechnology) according to the manufacturer’s protocol. RNA samples with A_260 nm_:A_280 nm_ above 1.8 were used for RT-PCR. cDNA was prepared using 50 ng of total RNA, oligo dT primer and PrimeScript RT Enzyme Mix (RT-for-PCR kit, Takara Biotechnology) according to the manufacturer’s instructions, in a total volume of 20 μL. Real-time PCR was performed on a CFX System (Bio-Rad). ∆CT values were determined by normalization to β-actin. Fold change values were calculated using the 2^−(∆∆CT)^ method. The primers used in this study are described in Table 4.

### Western blot

Homogenized liver tissue specimens were boiled with 2 × SDS-PAGE loading buffer at 95 °C for 10 min. Then, proteins were separated by 10% SDS-PAGE gel electrophoresis and transferred onto a PVDF membrane. After blocking with 5% nonfat dried milk in TBS-T (10 mM Tris-HCl, pH7.8, 150 mM NaCl and 0.05% [v/v] Tween-20) for 2 h at room temperature, the membrane was incubated with anti-TXNIP antibody (Cell Signaling Technology) or anti-β-actin antibody, diluted 2,000 to 5,000 fold, for at least 3 h at room temperature. The membrane was then washed and incubated with anti-rabbit secondary antibodies (5,000 to 10,000 dilution) for 1 h at room temperature. Protein bands were detected using the ChemiDoc XRS^+^ detection system (ECL, Bio-Rad); densitometric analysis was performed using Quantity One^®^ Image Analyzer (Bio-Rad).

### Statistical analysis

Data are mean ± standard error of the mean (SEM). Event incidence was compared using Fisher’s exact test. Differences of body weights were examined by two way ANOVA; the remaining parameters were evaluated by unpaired t test or one way ANOVA with *post hoc* Tukey’s test (Bartlett’s test for equal variances not significantly different at P > 0.05) or Dunnett’s multiple comparison test (otherwise). Graph Pad Software (San Diego, CA, USA) was used for statistical analyses. A P-value less than 0.05 was considered statistically significant.

## Additional Information

**How to cite this article**: Han, M. *et al.* Safety and anti-hyperglycemic efficacy of various tea types in mice. *Sci. Rep.* **6**, 31703; doi: 10.1038/srep31703 (2016).

## Supplementary Material

Supplementary Information

## Figures and Tables

**Table 1:** Catechin content of tea infusion (mg/mL).

Tea	GA	EGC	C	EC	GCG	ECG	EGCG	Total catethins
Green	0.07 ± 0.00	0.80 ± 0.01	0.17 ± 0.00	0.23 ± 0.02	0.15 ± 0.00	0.46 ± 0.04	2.52 ± 0.13	4.33 ± 0.02
Black	0.07 ± 0.01	0.02 ± 0.00	0.02 ± 0.00	0.01 ± 0.00	0.00 ± 0.00	0.01 ± 0.00	0.04 ± 0.00	0.10 ± 0.00
Yellow	0.10 ± 0.01	0.22 ± 0.00	0.09 ± 0.00	0.08 ± 0.01	0.17 ± 0.00	0.15 ± 0.00	0.59 ± 0.02	1.30 ± 0.00
Smoked	0.27 ± 0.00	0.54 ± 0.01	0.28 ± 0.01	0.22 ± 0.00	0.15 ± 0.00	0.30 ± 0.01	2.15 ± 0.06	3.64 ± 0.01

Total catechins represent the sum of epigallocatechin (EGC), catechin (C), epicatechin (EC), (−)-epigallocatechin-3-gallate (EGCG), (−)-gallocatechin-3-gallate (GCG) and (−)-epicatechin-3-gallate (ECG), not including gallic acid (GA). Data are presented as mean ± SEM (n = 2).

**Table 2:** Caffeine, theanine and sugar content of tea infusion (mg/mL).

Tea	Caffeine	Theanine	Soluble sugar	Polysaccharide
Green	1.08 ± 0.05	0.38 ± 0.02	0.69 ± 0.01	0.13 ± 0.00
Black	0.91 ± 0.02	0.24 ± 0.02	0.84 ± 0.01	0.28 ± 0.00
Yellow	0.59 ± 0.15	0.05 ± 0.00	1.49 ± 0.02	0.36 ± 0.00
Smoked	1.33 ± 0.03	0.55 ± 0.02	0.77 ± 0.01	0.16 ± 0.01

Data are presented as mean ± SEM (n = 2).

**Table 3:** Hepatic and gastrointestinal toxicities in mice.

Tea	Serum ALT (U/mL)	Serum AST (U/mL)	Serum LDH (U/mL)	Black intestine	Bad smell
Control	13.4 ± 2.3	14.4 ± 5.2	892 ± 198	0/7	0/7
Green	16.8 ± 3.4	14.9 ± 2.6	1032 ± 284	0/7	0/7
Black	18.7 ± 1.6	13.6 ± 2.9	1304 ± 83	0/6	0/6
Yellow	19.4 ± 2.7	19.8 ± 5.7	1359 ± 72	0/6	0/6
Smoked	70.1 ± 33.0*	54.3 ± 21.6*	3094 ± 738**	6/6***	5/6**

Mice were allowed free access to water (control group) or tea infusion (1:30, w/v), and regular diet for one week. Data are presented as mean ± SEM (n = 6 or 7). Compared to control, *P < 0.05, **P < 0.01 and ***P < 0.001.

**Table 4:** Primer sequences for RT-PCR.

Gene	Forward (5′−3′)	Reverse (5′−3′)
Actin beta	GCTGAGAGGGAAATCGTGCGT	ACCGCTCGTTGCCAATAGTGA
G6Pc	TTGCCAGGAAGAGAAAGAAGGAT	AACACAGACACAACTGAAGCCG
PEPCK1	AAAGCAAGACAGTCATCATCACCCA	TCTCAAAGTCCTCTTCCGACATCC
TXNIP	AATACCCCTGACCTAATGGCACC	ATTCGAGCAGAGACTGACACACG
Trx1	CCTTCTTCCATTCCCTCTGTGAC	TTTCCTTGTTAGCACCGGAGAAC
TrxR1	ACCTGGGCATCCCTGGAGAC	GCACCATTACAGTGACGTCTAAGC
ACCα	AGGAGGGAAAGGGATCAGAAAAG	CAGAGCAGTCACGACCAAACAAA
ACCβ	AGACACTGCAAATCCCAACCTTAC	CTTCGTCCACATCCTTCACACA
Foxo1	ATGGTGAAGAGCGTGCCCTACT	TCATTCTGCACTCGAATAAACTTGC
Srebp1	AGTCCAGCCTTTGAGGATAGCC	CCGTAGCATCAGAGGGAGTGAG
SCD1	TCCTCCTTGGATTGTGTAGAAACTT	AATGTCAGAAGAAATCAGGTGGGTA
FASN	CGTGTGACCGCCATCTATATCG	TGAGGTTGCTGTCGTCTGTAGTCTT
CPT1	TATGGTCAAGGTCTTCTCGGGTCG	AGTGCTGTCATGCGTTGGAAGTCTC
Acox1	TAAACACCCACCCACCAAGAAAG	CCTGGAGGTAAAGACAAGCAACT
