# Corrigendum: Safety and anti-hyperglycemic efficacy of various tea types in mice

**DOI:** 10.1038/srep35699

**Published:** 2016-11-08

**Authors:** Manman Han, Guangshan Zhao, Yijun Wang, Dongxu Wang, Feng Sun, Jingming Ning, Xiaochun Wan, Jinsong Zhang

Scientific Reports 6: Article number: 31703; 10.1038/srep31703 published online: 08 17 2016; updated: 11 08 2016.

The original version of this Article contained a typographical error in the spelling of the author Xiaochun Wan, which was incorrectly given as Xiachun Wan. This has now been corrected in the PDF and HTML versions of the Article.

