# Supplementary material for: Safety and anti-hyperglycemic efficacy of various tea types in mice
Source: Sci Rep. 2016 Aug 17;6:31703. doi: 10.1038/srep31703 (PMC4987693; doi:10.1038/srep31703)
Supplement: Supplementary Information [file srep31703-s1.pdf]

Supplementary Information

Safety and anti-hyperglycemic efficacy of various tea types in mice

Manman Han#, Guangshan Zhao#, Yijun Wang#, Dongxu Wang, Feng Sun, Jingming Ning, Xiachun Wan*, Jinsong Zhang*

State Key Laboratory of Tea Plant Biology and Utilization, School of Tea & Food Science, Anhui Agricultural University, Hefei, Anhui 230036, PR China

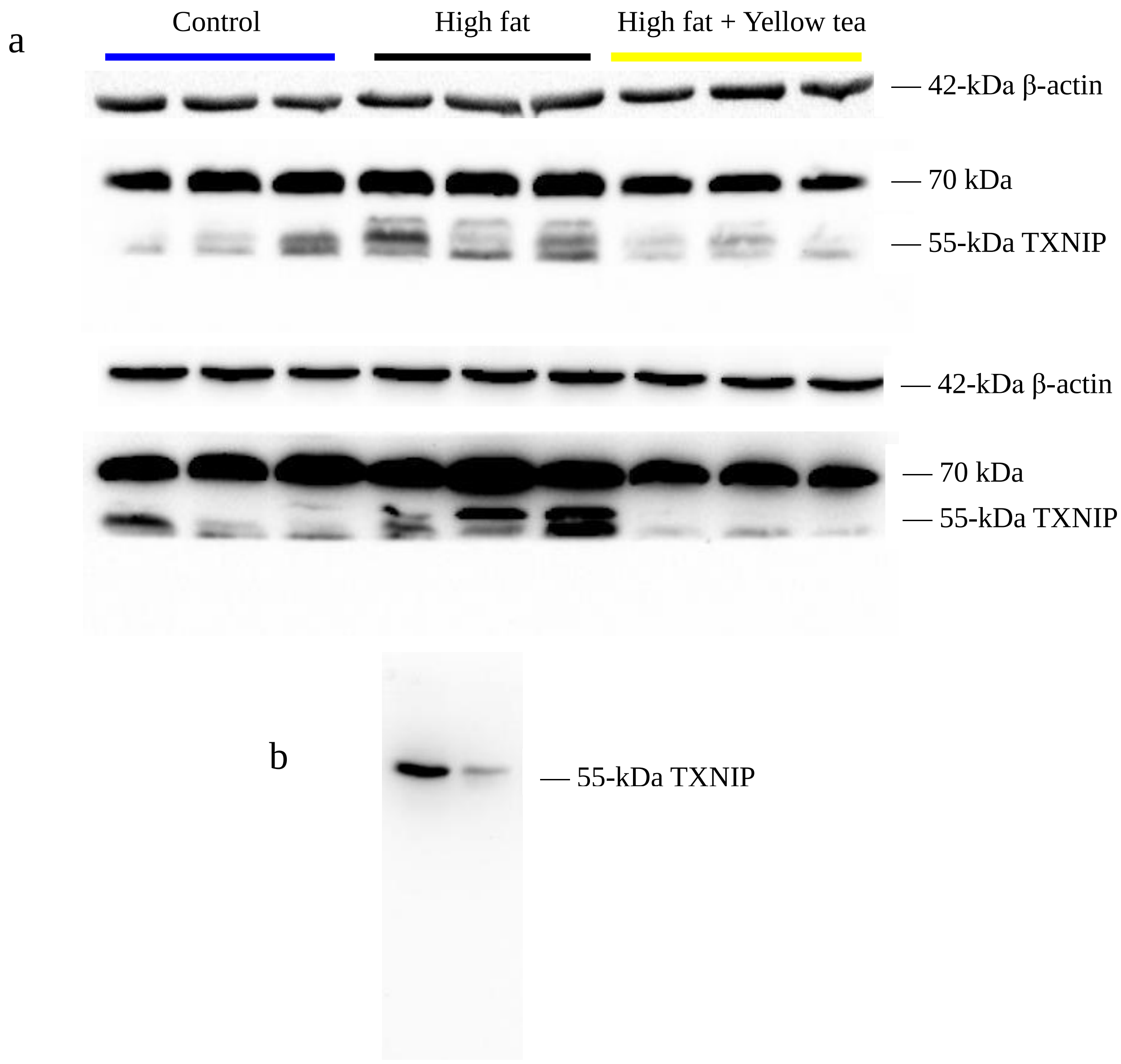

Supplementary Figure 1. Non-cropped blot for the experiment shown in Fig. 4b of the main article. Note: TXNIP antibody purchased from Cell Signaling Technology (#14715, which generates a specific binding with mouse TXNIP at 55 kDa) was used in this experiment. The nonspecific band at 70 kDa found in liver samples of ICR mice (a) did not exhibit in liver samples of healthy Kunming mice (b). Nonspecific band is not an unusual phenomenon, for example, an extremely strong nonspecific band at 40 kDa above the 27-kDa p27 (Supplemental Figure 5H, Sci Rep. 2016;6:28080) and huge nonspecific bands just below the 55-kDa ATF6 (Supplemental Figure 2C, Sci Rep. 2015;5: 10340).

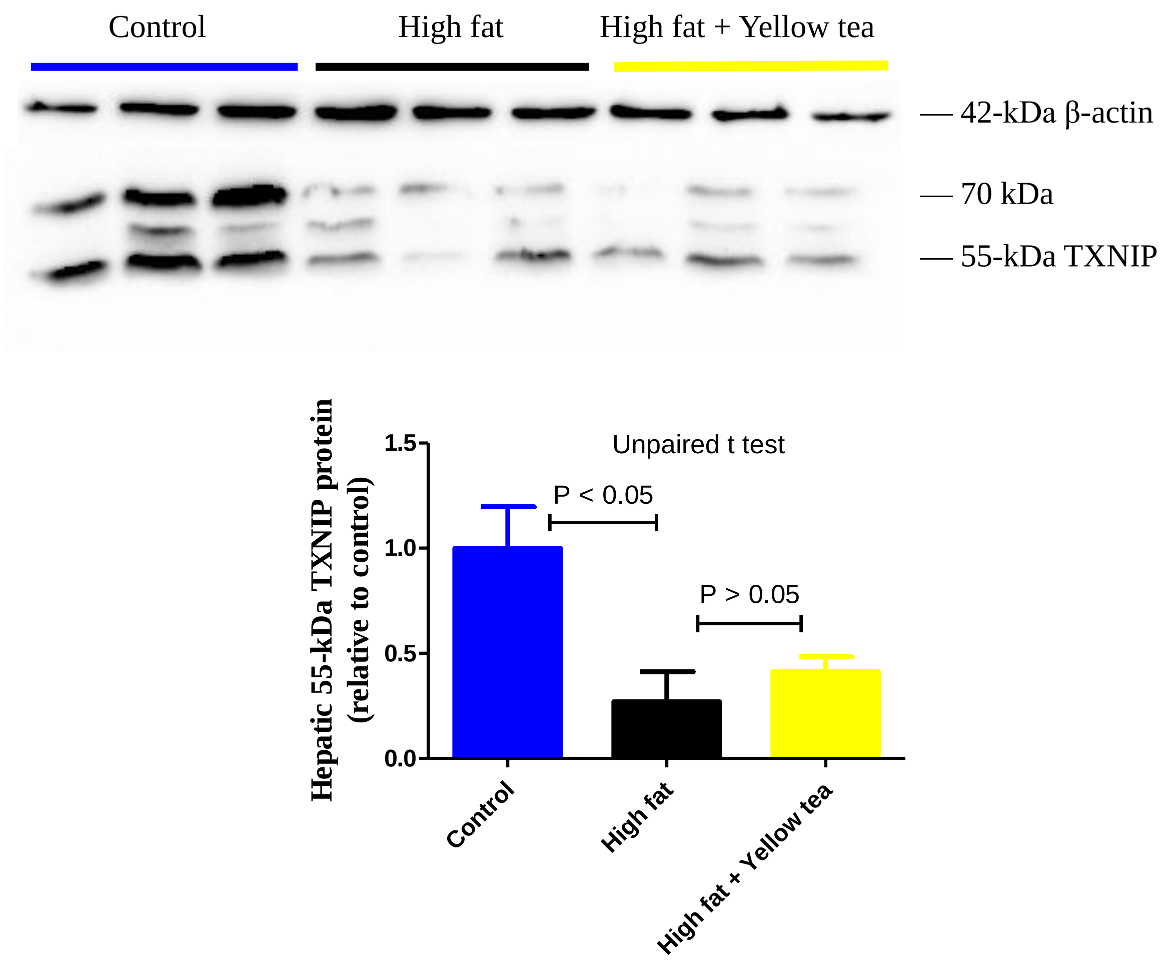

Supplementary Figure 2. Hepatic TXNIP protein (25-day high-fat feeding experiment). Mice were allowed free access to water and regular diet (control), water and high-fat diet as model (high fat) or yellow tea infusion (1:30, w/v) and high-fat diet for 25 days. Data are presented as mean ± SEM (n = 3).

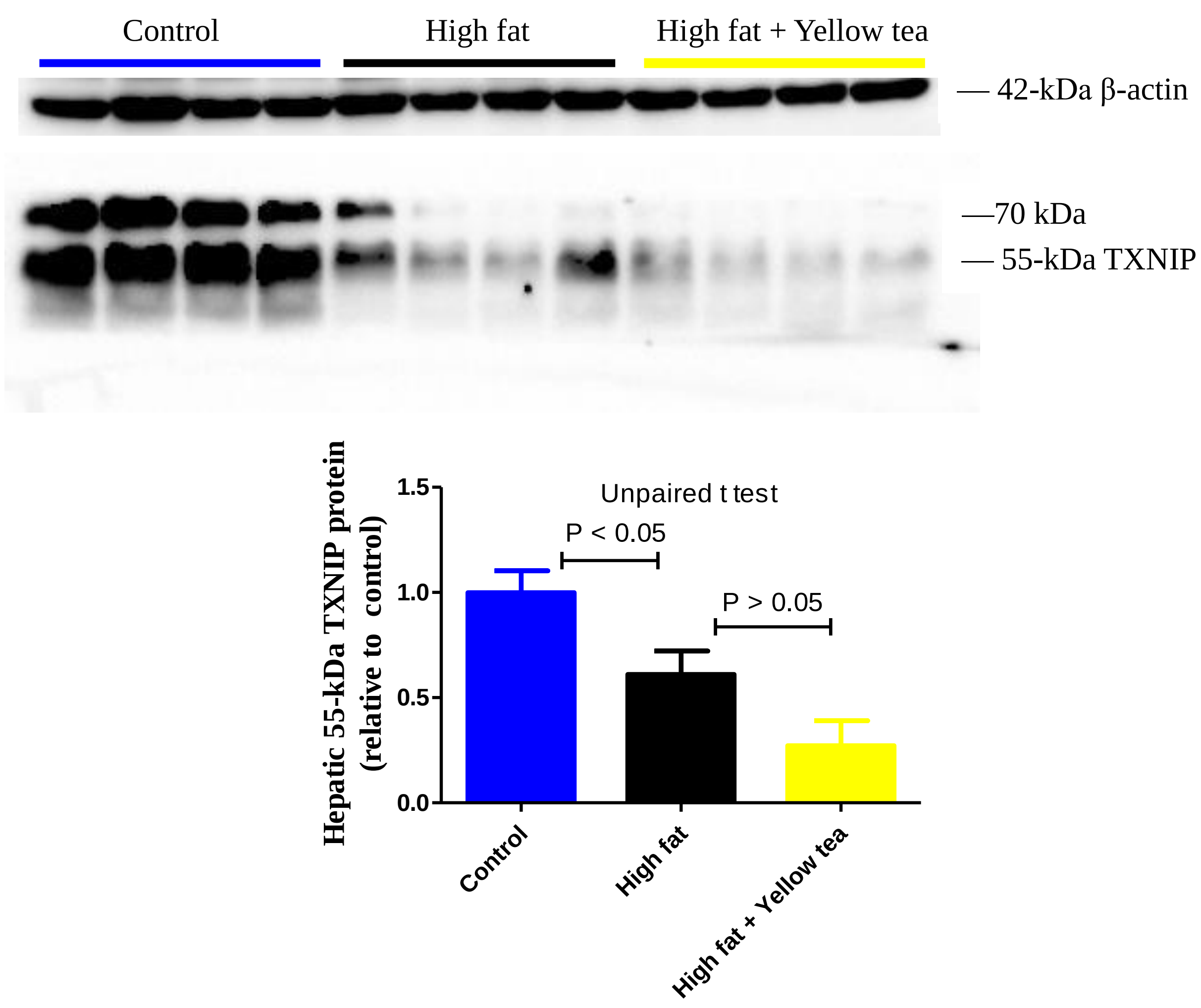

Supplementary Figure 3. Hepatic TXNIP protein (36-day high-fat feeding experiment). Mice were allowed free access to water and regular diet (control), water and high-fat diet as model (high fat) or yellow tea infusion (1:30, w/v) and high-fat diet for 36 days. Data are presented as mean ± SEM (n = 4).

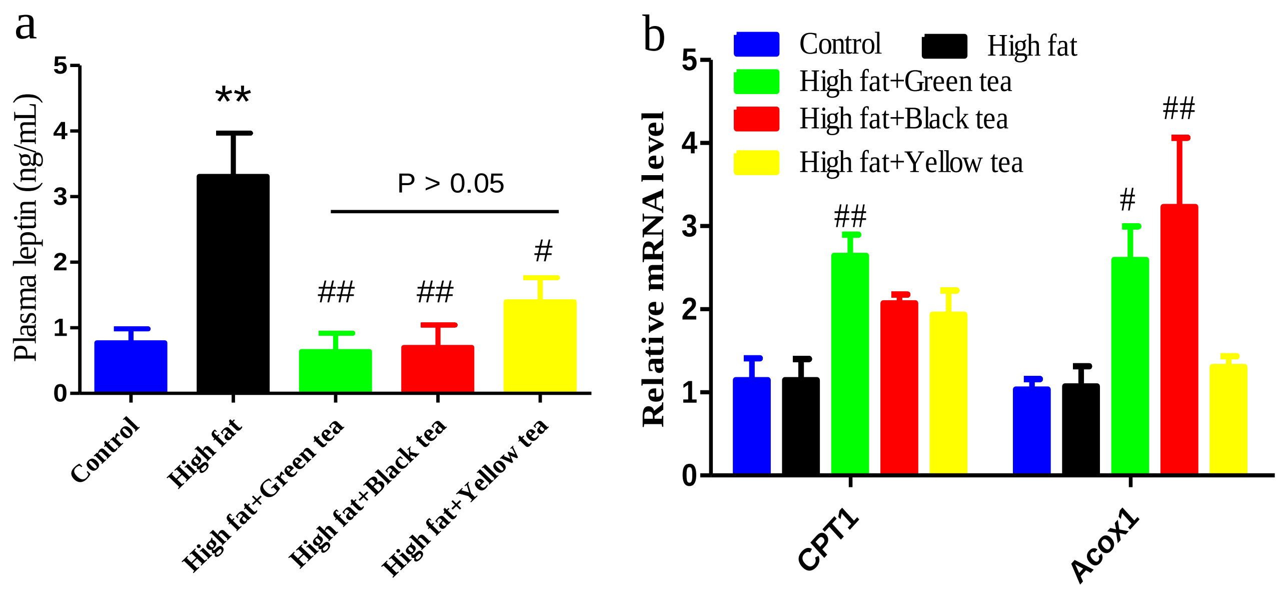

Supplementary Figure 4. Influence of tea infusion on plasma leptin and hepatic beta-oxidation genes. Mice were allowed free access to water and regular diet (control), water and high-fat diet as model (high fat) or tea infusion (1:30, w/v) and high-fat diet for 25 days. (a) Plasma leptin. (b) Hepatic genes of CPT1 and Acox1. **P < 0.01, compared to control. #P < 0.05 and ##P < 0.01, compared to high fat. Data are presented as mean ± SEM (n = 4-6).
